# Protocol for a multisite study on the efficacy of transcranial direct current stimulation as an adjuvant to naming and spelling therapy in the treatment of oral and written naming in individuals with primary progressive aphasia

**DOI:** 10.3389/fnhum.2025.1611272

**Published:** 2025-09-02

**Authors:** Michèle Masson-Trottier, Donna Tippett, Brenda Rapp, Denise Y. Harvey, Carlos Roncero, Leslie Vnenchak, Andreia Faria, Constantine Frangakis, Howard Chertkow, Roy H. Hamilton, Argye E. Hillis, Kyrana Tsapkini

**Affiliations:** ^1^Department of Neurology, Johns Hopkins School of Medicine, Baltimore, MD, United States; ^2^Department of Physical Medicine and Rehabilitation, Johns Hopkins University, Baltimore, MD, United States; ^3^Department of Otolaryngology—Head and Neck Surgery, Johns Hopkins University, Baltimore, MD, United States; ^4^Department of Cognitive Science, Johns Hopkins University, Baltimore, MD, United States; ^5^Department of Neurology, University of Pennsylvania, Philadelphia, PA, United States; ^6^Rotman Research Institute, Baycrest Health Sciences, Toronto, ON, Canada; ^7^Department of Radiology, Johns Hopkins Medicine, Baltimore, MD, United States; ^8^Department of Psychiatry and Behavioral Sciences, Johns Hopkins Medicine, Baltimore, MD, United States; ^9^Department of Biostatistics, Johns Hopkins School of Public Health, Baltimore, MD, United States; ^10^Department of Neurology and Neurosurgery, McGill University, Montreal, QC, Canada; ^11^Kimel Family Centre for Brain Health and Wellness and Anne & Allan Bank Centre for Clinical, Toronto, ON, Canada; ^12^Research Trials, Baycrest Health Sciences, Toronto, ON, Canada; ^13^Canadian Consortium on Neurodegeneration in Aging, Montreal, QC, Canada; ^14^Division of Neurology, Department of Medicine, University of Toronto, Toronto, ON, Canada; ^15^Lady Davis Institute for Medical Research, Jewish General Hospital, Montreal, QC, Canada

**Keywords:** primary progressive aphasia, language therapy, transcranial direct current stimulation, imaging biomarkers, molecular biomarkers, clinical trial

## Abstract

Primary progressive aphasia (PPA) is a neurological syndrome characterized by the gradual deterioration of language capabilities. Due to its neurodegenerative nature, PPA is marked by a continuous decline, necessitating ongoing and adaptive therapeutic interventions. Recent studies have demonstrated that behavioral therapies, particularly when combined with neuromodulation techniques such as transcranial direct current stimulation (tDCS), can improve treatment outcomes, including the long-term maintenance and generalization of therapeutic effects. However, there has yet to be a phase II multisite study examining the efficacy of tDCS in individuals with PPA. This paper reports the methods and analyses for the clinical trial NCT05386394. A total of 120 adults with non-fluent and logopenic variant PPA will receive a novel spoken Naming and Spelling (NaSp), individuals with semantic variant PPA will be excluded from this trial. Participants will receive NASP therapy over two periods of 3 weeks (Monday through Friday, for a total of 15 non-consecutive days) combined with anodal (a-tDCS) and sham tDCS (s-tDCS). They will be randomly allocated to receive a-tDCS either during the first or second intervention period. The study will be conducted at four sites across the United States and Canada. Outcome measures will be recorded immediately before and after each intervention period, as well as 3 months after each period. Primary outcome measures will be the change in phonemic accuracy in spoken picture naming and letter accuracy in spelling for trained nouns and verbs. Changes from the a-tDCS and s-tDCS periods will be compared to determine the efficacy of tDCS. Primary outcomes will be analyzed using statistical methods that account for repeated measures within participants (namely generalized estimating equations). A significant adjuvant effect of tDCS will be determined if differences in phonemic accuracy and/or letter accuracy immediately following a-tDCS intervention and/or at the 3-month follow-up are greater (at p < 0.05) than those of the s-tDCS intervention. This trial is the first multisite, fully powered, randomized, double-blind, sham-controlled, crossover study of the effectiveness of tDCS as an adjuvant to behavioral treatment for spoken naming and spelling deficits in individuals with PPA. Specific challenges in designing the protocol are considered.

## Introduction

### Prior research

#### Behavioral therapies

Throughout the progression of primary progressive aphasia (PPA), research supports the use of behavioral interventions targeting two complementary goals: (1) reducing impairments through restitutive therapies^1^ enhancing communication, and (2) increasing daily life participation^2^ through more compensatory approaches (Robinaugh and Henry, 2022; Taylor-Rubin et al., 2021). Restitutive therapies aim to restore lost functions and are typically adapted from well-established therapies for stroke-induced aphasia that target language skills, such as word finding, comprehension, and sentence production. Compensatory approaches focus on supporting individuals in finding alternative ways to accomplish tasks and enhance their active participation despite communication impairments.

Although therapies for PPA resemble those for stroke-induced aphasia, outcomes must be interpreted in light of progressive decline. Unlike stroke-induced aphasia, which results from a sudden, delimited brain injury and exhibits spontaneous recovery, PPA presents distinct challenges with its progressive language decline due to more diffuse neurodegeneration, even when receiving therapy (Norise and Hamilton, 2016). Therefore, a necessary consideration when measuring therapy efficacy in individuals with PPA is the expected gradual decline in abilities throughout the intervention period. Still, it remains crucial to assess the retention of skills present at baseline and the learning of newly acquired skills after the end of therapy (maintenance) and their generalization to other target words and language abilities (Cotelli et al., 2020). These measures are essential for understanding the functional significance of a given therapy and its impact on participants' daily communication. See Cotelli et al. (2020), Beales et al. (2018), and Cadorio et al. (2017) for reviews on maintenance and generalization following naming treatments with individuals living with PPA.

A recent systematic review identified 103 studies that reported behavioral treatments for individuals living with PPA (Wauters et al., 2023). Many studies were focused on restitutive therapies targeting anomia and agraphia since these are among the most common deficits in all variants. Anomia, for example, is a symptom encountered in all PPA variants. However, it may be caused by a breakdown at different levels of the naming process (e.g., at the conceptual level in individuals with semantic variant PPA (svPPA) and the lexical access level in individuals with logopenic PPA (lvPPA). Few studies included in the review investigated restitutive therapy targeting other areas of language and communication, such as semantic knowledge, phonology, speech production and fluency, syntax and morphology, word comprehension, spoken discourse production, or general language and cognition, and compensatory therapies such as augmentative and alternative communication and more functional communication strategies (Wauters et al., 2023).

Restitutive therapies targeting anomia in individuals living with PPA have yielded encouraging results immediately following intervention for trained items and generalization to untrained items, as well as leading to maintenance of therapy gains several weeks after therapy. Indeed, reviews supporting the benefits of spoken naming therapies have been published in recent years (Robinaugh and Henry, 2022; Taylor-Rubin et al., 2021; Wauters et al., 2023; Volkmer et al., 2020). Various approaches have been documented with individuals living with PPA, including semantic therapies emphasizing the meaning of the target words and phonological/orthographic therapies involving sound or spelling cues for the target words, for both nouns and verbs (Robinaugh and Henry, 2022; Taylor-Rubin et al., 2021; Wauters et al., 2023; Volkmer et al., 2020). These therapies enhance semantic or phonological activation to support word retrieval, following psycholinguistic models like the restricted interaction account (Goldrick and Rapp, 2002). Although maintenance and generalization are not measured in all studies, most of the high-quality studies identified in the review by Wauters et al. (2023) documented at least one participant exhibiting generalization and maintenance of treatment gains. The magnitude of therapy effects in these studies was small, as could be expected given the context of a neurodegenerative disorder.

Furthermore, restitutive behavioral therapies targeting agraphia in individuals living with PPA have also shown positive results as reported by recent reviews (Taylor-Rubin et al., 2021; Wauters et al., 2023), and more specifically, work done by our team (Rapp and Glucroft, 2009; Tsapkini and Hillis, 2013; Tsapkini et al., 2014, 2018). In an initial study, Tsapkini et al. (2014) implemented an intervention targeting the relearning of phoneme-to-grapheme conversion (PGC, also known as sound-to-letter correspondence), a skill that is often impaired in individuals with PPA. This intervention yielded gains for the trained phoneme-grapheme pairs (Tsapkini et al., 2014). In a subsequent trial, Tsapkini et al. (2018) applied the “spell-study-spell” therapy, representing a “lexical” therapy focusing on training the spelling of entire words (Rapp and Glucroft, 2009). Again, therapy led to improvements in spelling for the trained words; however, there was no generalization to untrained words when therapy was given alone (Tsapkini et al., 2018).

#### Neuromodulation

Neuromodulation methods, like tDCS, have attracted significant interest over the past decade as promising adjunctive interventions for individuals living with PPA. As recent studies and meta-analyses of combined behavior and neuromodulation interventions in individuals with PPA have shown, a great strength of these approaches (and tDCS in particular) is the increased maintenance and generalization effects compared to behavioral therapy alone, at least in spoken naming and spelling (Cotelli et al., 2020; Coemans et al., 2021; Wang et al., 2023; Nissim et al., 2020). tDCS involves safe, non-invasive application of electrical currents to modulate neuronal activity in targeted brain regions, enhancing neural plasticity (Norise and Hamilton, 2016; Rahman et al., 2013; Sebastian et al., 2016; Stagg and Nitsche, 2011) and potentially aiding language recovery in individuals with PPA by modulating cortical excitability (Ficek et al., 2018; Meinzer et al., 2015; Tao et al., 2021).

tDCS is thought to modulate neural activity by altering cortical excitability and neurotransmitter concentrations, particularly by subthreshold modulation of neuronal membranes (Stagg and Nitsche, 2011). In PPA, tDCS has been shown to modulate functional connectivity (FC), which can serve as an indirect correlate of cortical excitability. In a similar crossover, double-blinded, sham-controlled trial preceding the present one, members of our team found that a-tDCS reduced hyperconnectivity between the stimulated left IFG and functionally connected areas more than sham-tDCS, both paired with the same oral and written naming therapy. Furthermore, these reductions correlated with improved language outcomes up to 2 months post-treatment (Ficek et al., 2018; Tao et al., 2021). These decreases usually reflect increased task efficiency as behavior becomes more automated and have been linked to better treatment outcomes (Meinzer et al., 2015). Notably, both reductions and increases in FC have been observed depending on baseline connectivity patterns, suggesting that tDCS may normalize by downregulating or upregulating either hyper- or hypoconnectivity, respectively, depending on the baseline, to support language recovery (Tao et al., 2023). Additionally, reductions in the inhibitory neurotransmitter GABA following tDCS have also been linked to behavioral gains in individuals with PPA (Harris A. D. et al., 2019). Emerging evidence points to variant-specific changes in FC and metabolic alterations induced by tDCS, as measured by FDG PET, extending mechanistic understanding of how tDCS supports recovery in PPA (Tao et al., 2023; Roncero et al., 2023).

Compared to other neuromodulation methods, such as transcranial magnetic stimulation (TMS), tDCS is significantly low-cost and easily portable, making it attractive to implement in home-based therapies (Tippett et al., 2024; Im et al., 2019; Cappon et al., 2024), a feasible and efficacious approach in individuals with PPA, in particular (Neophytou et al., 2024). The simultaneous use of tDCS with behavioral language therapies aligns with the current understanding of neuromodulation mechanisms: the effects of stimulation on cortical reorganization appear to be influenced by the individual's state or current effort, with behavioral activity during or near the time of stimulation directly impacting outcomes (Silvanto et al., 2008; Gill et al., 2015). Research suggests that maximum benefits from stimulation are obtained when the brain area is actively engaged in a relevant behavioral task during or near the time of stimulation (Bikson and Rahman, 2013). The specificity of tDCS has also been demonstrated in PPA, as generalization of tDCS effects occurred only for functions related to the stimulated area (Wang et al., 2023).

Different PPA variants are associated with distinct patterns of neurodegeneration that affect language processing. For example, individuals living with lvPPA typically show predominant left posterior perisylvian or parietal atrophy, while the neurodegeneration in individuals living with non-fluent variant PPA (nfvPPA) is marked by predominant left posterior fronto-insular atrophy (Gorno-Tempini et al., 2011). These regions are critically involved in phonological working memory, syntactic processing, and motor speech planning. Individuals living with svPPA display distinct predominant anterior temporal lobe atrophy and semantic impairments (Gorno-Tempini et al., 2011). This study focuses on lvPPA and nfvPPA, where stimulation of the left inferior frontal gyrus (IFG) is theoretically and anatomically relevant. Given the importance of the left IFG across various language functions, this area has become a key target for neuromodulation in tDCS studies aimed at enhancing language outcomes in PPA (Cotelli et al., 2020; Nissim et al., 2020).

For over 10 years, the research teams involved in this multisite study have investigated the potential of tDCS as a catalyst for therapeutic interventions. In the same studies discussed previously, Tsapkini et al. (2014, 2018) investigated the effectiveness of tDCS paired with speech-language treatment in individuals with PPA through randomized, double-blind, sham-controlled, within-subject crossover designs. In these studies, anodal-tDCS (a-tDCS) was applied over the left IFG simultaneously with the written naming/spelling therapies (Tsapkini et al., 2014; 2018). These studies produced positive outcomes with a-tDCS combined with behavioral therapy, yielding over 15% more improvement than behavioral therapy paired with sham-tDCS (s-tDCS) for individuals with PPA, albeit at different degrees for each variant. Significantly, a-tDCS promoted the generalization of treatment effects to untrained items and beyond the targeted tasks to functions subserved by the stimulated area (Tsapkini et al., 2018; Wang et al., 2023). Studies by Roncero et al. (2017, 2019) have found similar results following left inferior parieto-temporal stimulation combined with spoken naming therapy: a-tDCS, when combined with behavioral therapy, led to over 20% more improvement than s-tDCS both paired with the same behavioral therapy, and to the generalization of treatment effects to untrained items. Other research groups have also reported that a-tDCS interventions lead to greater maintenance of gains over time compared to behavioral therapies alone, highlighting the potential of this non-invasive neuromodulation technique as an adjunct to behavioral therapies in the management of PPA. As research in this area continues to evolve, tDCS offers new possibilities for improving language outcomes and quality of life in individuals living with PPA (Cotelli et al., 2020; Coemans et al., 2021; Nissim et al., 2020; Sebastian et al., 2016; Byeon, 2020).

Predicting therapy outcomes in individuals living with PPA remains a complex endeavor, with numerous factors influencing treatment response. Factors such as the PPA variant have been shown to influence therapy response. For example, it was found that those with non-fluent variant PPA (nfvPPA) tend to benefit more from tDCS (i.e., improve in both trained and untrained material) (Tsapkini et al., 2018). Moreover, individuals living with either nfvPPA or lvPPA have shown good therapy response and generalization (Tsapkini et al., 2018), contrasting with individuals with svPPA for whom comprehension deficits could limit generalization (Tsapkini et al., 2018; Rogalski et al., 2011), though they are still able to learn (Tsapkini et al., 2018; Hung et al., 2017). Despite strides in identifying a variety of predictors, including structural imaging (brain region volumes and cortical thickness) (de Aguiar et al., 2020a; Nissim et al., 2022), white-matter integrity (Zhao et al., 2021), functional connectivity (Wang D. et al., 2024), baseline language and cognitive abilities (de Aguiar et al., 2020b; McConathey et al., 2017), demographic (e.g., sex; see Licata, Zhao (Licata et al., 2023) and lifestyle factors (e.g., sleep (Herrmann et al., 2022), gaps persist in predicting therapy outcomes and tDCS responsiveness. It is not fully understood which factors predict the effectiveness of tDCS more than others. In this regard, genetic factors also appear to play a significant role.

#### Genetic factors

Individual differences in genetic makeup may play a significant role in shaping the efficacy of neuromodulatory interventions such as tDCS. Genetic factors that influence synaptic plasticity, neurotransmitter function, or vulnerability to neurodegeneration are hypothesized to interact with tDCS-induced neuroplasticity. Although this interaction remains untested in PPA (Wang Z. et al., 2024), related evidence from other neurodegenerative disorders, post-stroke aphasia, and healthy aging provides a compelling theoretical basis for examining genetic predictors in this context.

The brain-derived neurotrophic factor (BDNF) gene plays a central role in regulating synaptic plasticity, neuronal survival, and long-term potentiation and fluctuations in its levels are associated with neuropathological changes observed in neurodegenerative disorders (see Azman and Zakaria, 2022; Ibrahim et al., 2022) for reviews on the molecular mechanism of action). A common single-nucleotide polymorphism, Val66Met (rs6265), is associated with decreased activity-dependent secretion of BDNF (Lamb et al., 2015) which may impair learning and memory; however, this finding is not supported by Bugge Kambestad et al. (2023). Structurally, individuals with the Val66Met gene show smaller hippocampal volume (Molendijk et al., 2012; Hajek et al., 2012; Kambeitz et al., 2012) and cortical thinning in regions relevant to PPA, such as the left frontal and temporal lobes (Yang et al., 2012), potentially impacting their responsiveness to tDCS-based interventions. Functionally, the Val66Met genotype is associated with greater decline in episodic memory and learning impairments (Lim et al., 2021; Cechova et al., 2020; Thomson et al., 2024). In individuals living with post-stroke aphasia, BDNF polymorphism has been linked to aphasia severity and stimulation-induced neuroplasticity (Dresang et al., 2022). In an RCT involving tDCS and language therapy in individuals living with post-stroke aphasia, BDNF val/val carriers showed greater benefit from a-tDCS and aphasia therapy than val/met carriers (Fridriksson et al., 2018). However, this advantage was not observed in a study of aphasia recovery without neuromodulation (de Boer et al., 2017).

The catechol-O-methyltransferase (COMT) gene encodes the COMT enzyme, which plays a crucial role in the degradation of dopamine, particularly in the prefrontal cortex. The Val158Met polymorphism affects enzymatic activity, with Met carriers exhibiting lower COMT activity and thus higher extracellular dopamine levels (Schacht, 2016), which may confer advantages in certain cognitive tasks but can also lead to inefficiencies under high-load conditions. The COMPT effect may be modulated by sex (Elton et al., 2017; Fan et al., 2020). In cognitively unimpaired older adults, COMT Val carriers benefited significantly from tDCS more than those with met/met genes (Hayek et al., 2021).

Finally, the APOE4 allele, particularly in its homozygous form, is a well-established genetic risk factor for Alzheimer's disease (AD), influencing Aβ plaque formation, tau aggregation, and synaptic loss (Sun et al., 2023). It has also been implicated in altered neuroplasticity and cognitive function, even in persons without AD (Corbo et al., 2023; Zimmermann et al., 2019). In the context of PPA, APOE4 is associated with increased β-amyloid deposition and disrupted neural connectivity, contributing to the disease's pathophysiology (Singh et al., 2023). However, its effects are heterogeneous across different phenotypes and subtypes of the disease. Homozygosity is linked to more severe disease progression and potentially poorer treatment response. Moreover, emerging evidence suggests that the APOE4 genotype may influence the effectiveness of tDCS. A recent study by Kang et al. (2021) found that cognitive remediation combined with tDCS slowed decline, with significantly greater benefits observed in APOE4 non-carriers. Understanding the specific pathways affected by APOE4 can guide the development of targeted interventions that may mitigate its adverse effects on PPA progression.

Taken together, BDNF, COMT, and APOE genotypes represent biologically plausible moderators of tDCS efficacy, influencing structural integrity, excitatory-inhibitory balance, and neuroplastic capacity. The present trial is the first to investigate these interactions in PPA and may help identify genetic profiles associated with enhanced responsiveness to neuromodulatory therapies.

#### Blood biomarkers

The role of biomarkers in relation to tDCS outcomes in individuals living with PPA remains largely unexplored, though several markers may offer insight into disease progression and therapy response. Neurofilament light chain (NfL), a neuronal cytoskeletal protein that plays a crucial role in maintaining the structural integrity of axons, is recognized as a valuable biomarker in the context of neurodegenerative disorders. It is released during neuroaxonal damage, making it a sensitive-but-not-specific biomarker for neurodegenerative processes (Matias-Guiu et al., 2019; Steinacker et al., 2017; Lin et al., 2019). The impact of NfL on therapy response in individuals living with PPA, however, remains unknown.

Additionally, tau is a microtubule-associated protein primarily found in neurons, which, when abnormally phosphorylated or fragmented, can aggregate and contribute to the development of neurodegenerative disorders. It can be an important biomarker in PPAs associated with tauopathies (Shir et al., 2024), and its presence in biological fluids may offer insights into disease progression and treatment efficacy, enhancing clinical utility.

Finally, the impact of amyloid-beta 42 (Aβ42) on language therapy efficacy in PPA is complex, encompassing neurodegeneration and therapeutic strategies. In individuals with lvPPA, the presence of Aβ42, signifying Alzheimer's disease, has been found to correlate with increased cortical atrophy, especially in the left hemisphere, and language deterioration, potentially influencing therapy results (Rogalski et al., 2019). This left hemisphere predominant atrophy correlates with a more significant decline in language performance, potentially reducing the efficacy of language therapy (Rogalski et al., 2019). To our knowledge, there has been no research exploring the interaction between Aβ pathology and neural mechanisms to better understand their combined impact on treatment efficacy in PPA. Together, these biomarkers could eventually inform individualized prognoses or treatment decisions; however, their role in moderating or mediating response to tDCS remains an open and important area for future investigation.

#### Lifelong experiences and lifestyle

Similar to genetic factors and biomarkers, lifelong language experiences, such as bilingualism, and lifestyle factors, including exercise and sleep habits, have been argued to influence cognitive reserve and neural plasticity, thereby affecting the efficacy of therapy. For example, bilingual individuals with post-stroke aphasia have been shown to benefit more from therapy and have better recovery than their monolingual peers (Masson-Trottier et al., 2022; Lahiri et al., 2021). However, to our knowledge, bilingualism in individuals living with PPA has not been investigated as a predictor of therapy outcomes (Norhan et al., 2023). Exercise has also proven to maintain and potentially improve cognitive functions in aging (Krivanek et al., 2021) and individuals with post-stroke aphasia (Lorenzen and Murray, 2008). Understanding the interplay between these factors and therapy outcomes could provide valuable insights for optimizing treatment approaches and enhancing clinical care for individuals with PPA.

In summary, studies focusing on behavioral treatment of common symptoms in individuals living with PPA, such as anomia and agraphia, have shown promising results in terms of improvements in trained items immediately following intervention, as well as generalization to untrained items and retention of therapy gains weeks after the intervention ends (Robinaugh and Henry, 2022; Taylor-Rubin et al., 2021; Cadorio et al., 2017; Wauters et al., 2023). Neuromodulation methods, especially tDCS, have further enhanced these outcomes, promoting the maintenance and generalization of language behaviors (Cotelli et al., 2020; Coemans et al., 2021; Nissim et al., 2020). However, a Phase II multisite study has yet to be performed to evaluate large-scale efficacy and further assess safety, examining the benefits of tDCS in individuals with PPA. Furthermore, predicting therapy outcomes in PPA remains complex, influenced by factors such as PPA variant, neuroanatomical and neurofunctional factors, and baseline cognitive abilities. Significant gaps remain in understanding the roles of genetic factors, biomarkers, and lifelong language experiences in shaping therapeutic outcomes, particularly with tDCS. Addressing these gaps is important for optimizing treatment approaches and improving clinical care and neuromodulation outcomes for individuals with PPA.

Despite promising findings from the previous single-site Phase II study using tDCS paired with similar language therapy in PPA (Tsapkini et al., 2018) and other studies from members of our group (Roncero et al., 2017, 2019; Hung et al., 2017; Nissim et al., 2022; McConathey et al., 2017), no multisite Phase II trial has systematically evaluated its efficacy, which is a helpful step to facilitate transfer of tDCS into the clinical environment. This gap is significant given the encouraging learning and generalization effects observed in lvPPA and nfvPPA populations. The present study is designed to address this gap by evaluating the impact of combined tDCS and behavioral therapy on naming and spelling in a well-powered multisite randomized controlled trial (RCT), and by identifying clinical, neural, and genetic predictors of treatment response. A crossover RCT enables each participant to serve as their own control, increasing power and reducing inter-individual variability.

### Objectives

This paper reports the protocol and analysis plan for the clinical trial NCT05386394, registered on http://clinicaltrials.gov, which implements a multisite, randomized, double-blind, sham-controlled, within-subject crossover design. The trial aims to assess the effectiveness of a-tDCS targeting the left IFG combined with a novel, theoretically driven behavioral naming treatment in improving spoken naming and spelling abilities in individuals with lvPPA or nfvPPA. The overall goal is to bring tDCS combined with spoken naming and spelling therapy from the research lab closer to the clinic. We hypothesize that a-tDCS targeting the left IFG concurrent with spoken naming and spelling therapy will yield superior improvements compared to sham-tDCS (s-tDCS) paired with the same behavioral therapy, with sustained effects for up to 3 months post-treatment and generalization to untrained items as well as broader language and cognitive tasks subserved by the same area of stimulation. Additionally, we aim to evaluate factors already identified as potential predictors of treatment response, including PPA subtype, aphasia severity, cortical and neurofunctional measures, cognitive abilities, genetic markers, lifelong language experience, and demographic variables such as age and sex.

## Methods and analysis

Table 1 provides the Standard Protocol Items: Recommendations for Interventional Trials (SPIRIT) details for the clinical trial regarding enrollment, interventions, and assessments (Chan et al., 2013).

**Table 1:**
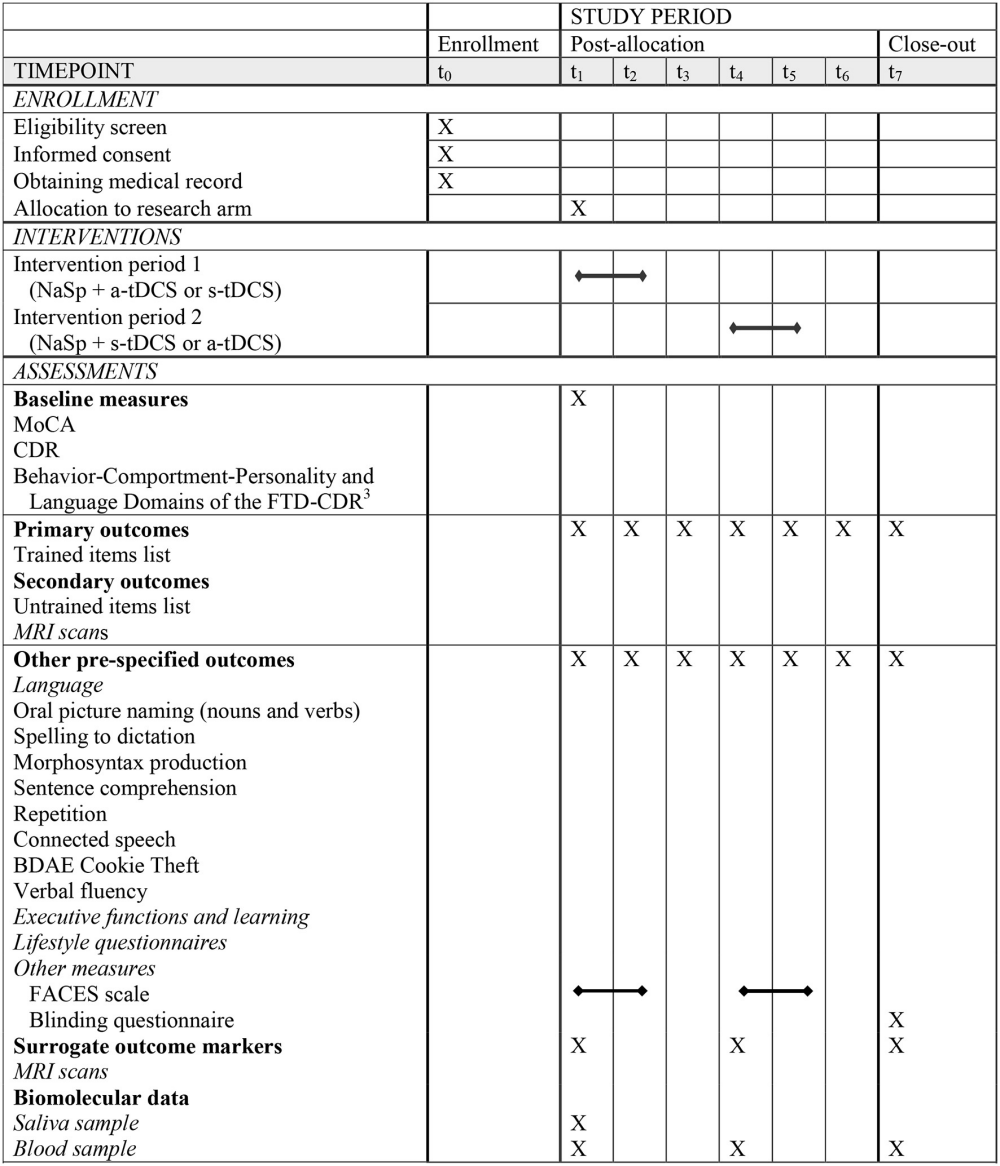
Standard Protocol Items: Recommendations for Interventional Trials (SPIRIT) details for the schedule of enrollment, interventions, and assessments, summarized version.

See Supplementary material for the detailed version of this table.

Interventions: NaSp, Naming and Spelling therapy; a-tDCS, active-transcranial direct current stimulation; s-tDCS, sham-transcranial direct current stimulation.

Assessments: Behavior-Comportment-Personality and Language Domains of the Frontotemporal Lobar Degeneration-Modified Clinical Dementia Rating (FTLD-CDR) (Knopman et al., 2008), Clinical Dementia Rating Scale (CDR) (Morris, 1993), Montreal Cognitive Assessment (MoCA) (Nasreddine et al., 2005).

### Trial design and settings

**Figure 1:**
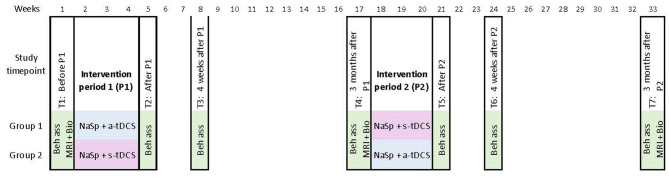
Time course of the study (15 NaSp intervention sessions per period) and evaluations for each group of participants in the crossover design. Beh ass, behavioral assessment; Bio, Biomolecular data; MRI, MRI scan; NaSp, Naming ans Spelling therapy; a-tDCS, active-transcranial direct current stimulation; s-tDCS, sham-transcranial direct current stimulation.

Building on a previous trial led by members of our team (NCT02606422), this study will employ a randomized, double-blind, sham-controlled, within-subject crossover design. Participants will be randomly assigned to one of two crossover groups (see Figure 1 for a visual representation of the timeline). Group 1 will receive a-tDCS over the left IFG, combined with Naming and Spelling therapy (NaSp), during Intervention Period 1, and s-tDCS over the left IFG, combined with NaSp, during Intervention Period 2. Conversely, Group 2 will receive NaSp with s-tDCS over the left IFG during intervention Period 1 and NaSp with a-tDCS over the left IFG during intervention Period 2.

Participants will be recruited through referrals from their neurologist, FTD, or AD research centers at each university, self-referrals via clinicaltrials.org, and in-house participant banks. This multi-site study is conducted across four research settings: Johns Hopkins School of Medicine, University of Pennsylvania, Baycrest Academy of Health Sciences and Geriatric Research, and Lady Davis Research Institute. All assessments and therapy sessions will be in person at the research facility. Participants will not receive these interventions in their clinical care context.

### Data collection

Demographic characteristics will be collected at the enrollment session (T0) after obtaining informed consent and the eligibility screening. Medical records will also be collected at that point. Saliva samples will be collected at baseline (T1) and blood samples will be collected before P1 (T1), at 3 months after P1 [i.e., before P2 (T4)], and at 3 months after P2 (T7).

Each participant will undergo clinical, cognitive, and language assessments at seven time points throughout the study: before intervention period 1 (P1) (T1), after P1 (T2), 4 weeks after P1 (T3), and 3-month after P1 [T4, also serving as before intervention period 2 (P2)], after P2 (T5), 8 weeks after P2 (T6), and at 3-month after P2 (T7). Figure 1 provides an overview of the study time course. Primary and secondary outcome measures will be consistently assessed for each participant, and the assessor will remain blinded to the participant's treatment allocation throughout all visits. Surrogate outcomes [Magnetic Resonance Imaging (MRI) scans] will be obtained before P1 (T1), at 3 months after P1 [i.e., before P2 (T4)], and at 3 months after P2 (T7).

### Sample size

Based on the crossover design, we calculated that 120 participants with PPA (40 at each of the three sites) are needed to achieve 85% power in detecting the target effect size (e.s.^target^). The power of the design to detect e.s.^target^ using an estimator is calculated as/is Φ(*e.s*_*target*_/√(*rate*_*design*_/*n*) - 1.96), where *rate*_*design*_/*n* represents the standard error squared of the estimator, and Φ is the cumulative distribution of the normal. Plausible values of *rate*_*design*_ and effect sizes are determined based on similar outcomes from previous studies with comparable crossover designs. The estimated 15% dropout rate is based on previous studies involving individuals with PPA undergoing tDCS and language therapy. In prior RCT from members of our team, attrition was ~15% during the first phase, mostly due to unrelated medical issues (Tsapkini et al., 2018). This rate was used to estimate the required sample size.

### Selection and treatment of participants

This study population will consist of 120 individuals diagnosed with lvPPA or nfvPPA by their neurologist, based on a comprehensive neurologic evaluation, with the diagnosis confirmed through neuroimaging. Based on previous research carried out by team members (Tsapkini et al., 2018), it was decided not to include individuals with svPPA because although they showed learning effects for trained items, the effects did not generalize to untrained items or tasks. Thus, this study aims to determine the adjuvant effect of tDCS in 2 populations of individuals living with PPA that responded to a similar type of therapy in our previously published studies.

Recruitment will also include 60 healthy controls matched with participants for age and education across all sites. These controls will participate in one MRI scan and one clinical, cognitive, and language assessment to evaluate the normal variability of the measures included in the trial and whether tDCS can normalize participants' neural response patterns.

### Randomization and blinding

As shown in Table 1, following the T1 assessment, participants will be randomly allocated to either Group 1 or Group 2, thereby determining the order of stimulation conditions. Randomization is performed independently for each variant (lvPPA and nfvPPA), employing block randomization with blocks of four participants, and each site performs randomization independently. Within each block, two individuals will be assigned to Group 1 and the other two to Group 2, ensuring balanced allocation across treatment arms within each PPA variant. Randomization ensures a balanced allocation of treatment sequences across participants, thereby controlling for potential order effects and minimizing biases associated with the crossover design (such as period effects and potential carry-over effects, although limited in this study by the washout period of 3 months (Li et al., 2015; Piantadosi, 2024).

Following the allocation, each participant will be assigned 15 unique five-digit codes provided by the manufacturer of the tDCS stimulator upon device programming (one for each intervention day), indicating whether they receive active stimulation (a-tDCS) or placebo (s-tDCS). The codes are necessary to initiate the tDCS delivery device, but do not reveal whether active or sham stimulation is administered. The research team (including therapists and assessors) and participants will be blinded to whether a- or s-tDCS is being administered during each period to maintain a fully blinded study design. The code list will be decrypted upon study completion to identify when each participant received a- and s-tDCS. The specific programming of the tDCS device during the s-tDCS period condition can be found in the tDCS section below.

### Inclusion and exclusion criteria

#### Inclusion criteria

We will include 120 participants with PPA who are between 50 and 80 years of age, right-handed, proficient in English, and have a minimum of a high school education. Participants must be diagnosed from a PPA and Alzheimer's disease (AD) clinics at the University of Pennsylvania, Johns Hopkins University, the University of Toronto, or other specialized referral centers in the US and Canada, using current consensus criteria (Gorno-Tempini et al., 2011). To corroborate the diagnosis, AD biomarkers (CSF or PET amyloid β I-42) will be examined and we will use our variant classification tests developed, including a spelling task and a speech production task (Cookie Theft picture description task (Goodglass and Kaplan, 1983), which can accurately discriminate PPA participants with over 80% accuracy (Themistocleous et al., 2021; Neophytou et al., 2019).

Participants are asked to maintain their daily habits as consistently as possible throughout the trial. Therefore, individuals already receiving speech-language therapy before enrolling in the project may continue to see their therapist. However, participants who were not already seeing a therapist are requested to wait until the end of the trial before starting any new therapy sessions.

#### Exclusion criteria

We will exclude individuals who have any of the following: a history of neurological disease, including vascular dementia, large vessel stroke, or attentional deficit; uncorrected hearing loss or visual acuity loss; a history of major pre-morbid psychiatric disorders; left-handedness; or advanced dementia impairments [Mini-Mental State Examination (MMSE) (Folstein et al., 1975) < 15] or severe language impairments preventing the participation in assessments and therapy.

### Interventional methods

#### NaSp therapy

In this trial, the overarching aim of the intervention is to enhance communication effectiveness in individuals living with PPA by improving their spoken naming and spelling of nouns and verbs. NaSp was developed based on our previous work (Tsapkini et al., 2014, 2018), and improved based on additional evidence from the literature to enhance spoken naming specifically (Rapp and Glucroft, 2009; Tsapkini and Hillis, 2013; Beeson et al., 2002). While NaSp includes elements from these earlier therapies, it also incorporates modifications that target spoken naming abilities—an aspect not effectively addressed by the therapy used in our prior studies.

Using the Rehabilitation Treatment Specification System (Hart et al., 2019) as a framework, NaSp can be described as a multi-stage intervention that is decomposable into *therapy targets*, each of which is linked to specific *active ingredients*. Specific *Skills and Habits* targets include improving spoken naming and spelling accuracy of trained and untrained nouns and verbs, increasing metacognitive skills to compensate for anomia, and enhancing language and cognitive skills to support communication. An *Organ Functions* target is to improve the efficiency of the language network in the brain in the presence of neurodegeneration. Finally, *Representation* targets are to increase confidence in communication and maximize engagement and collaboration. While breaking NaSp down into its specific active ingredients provides a detailed therapeutic perspective, it goes beyond the scope of this paper and will be addressed in a subsequent publication.

**Figure 2:**
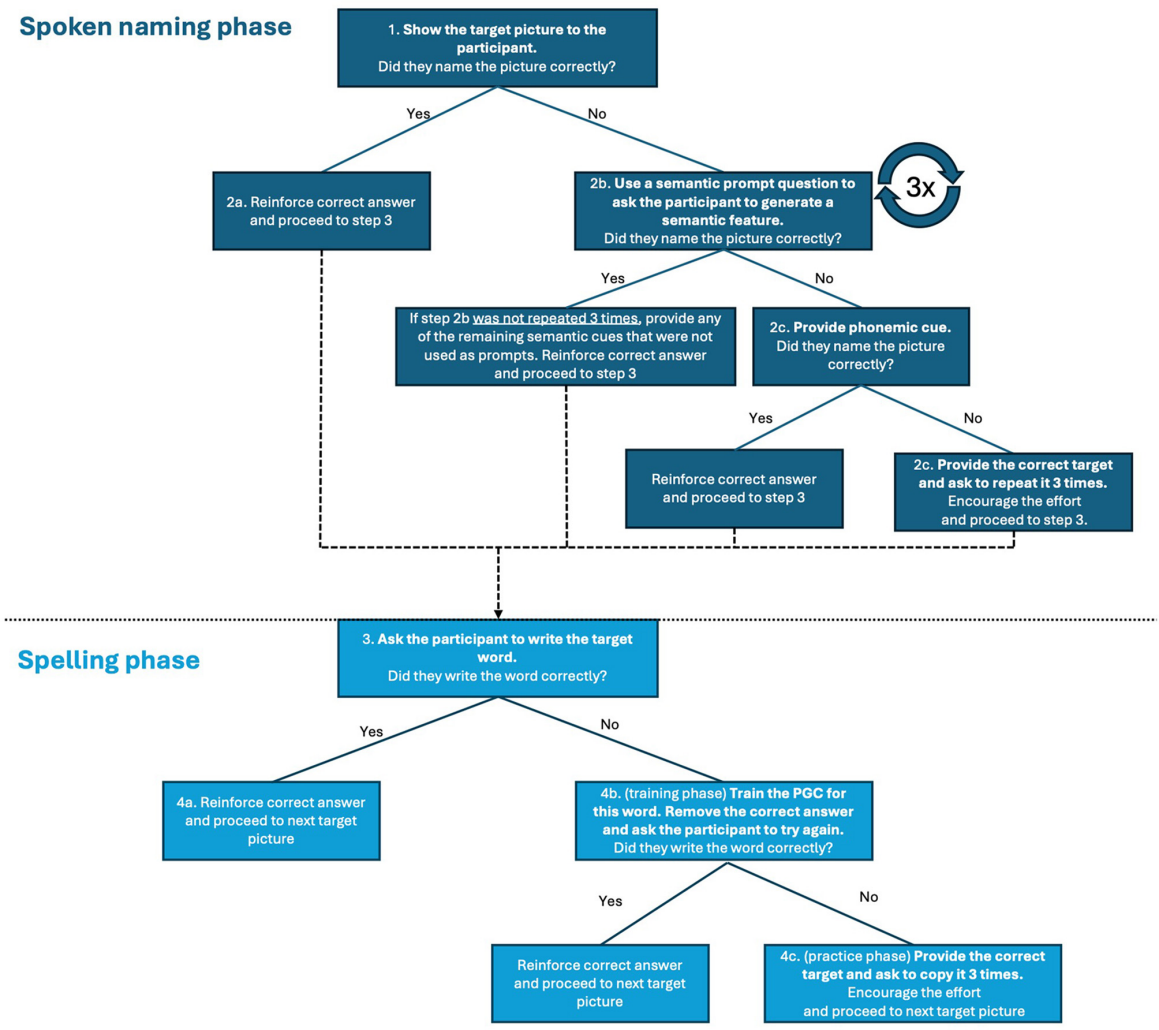
The flow of NaSp therapy.

During NaSp therapy sessions, each trial consists of two phases –spoken naming and spelling. Figure 2 illustrates the flow of NaSp therapy. Each trial begins with the spoken naming phase, in which individuals with PPA are presented with a picture and asked to name it orally. If successful, they proceed to write the word. If unsuccessful, they receive prompts to generate three semantic cues to aid in naming, followed by a phonemic cue if needed. During the cueing, participants are encouraged to name the picture as early as they can (i.e., they do not need to wait for the three semantic cues). If the participant can still not name the picture, the spoken word is provided, and they are asked to repeat it three times before moving on to spelling. In the spelling phase, participants are asked to write the word corresponding to the picture. Successful attempts are reinforced by confirming the provided answer is correct, while unsuccessful attempts prompt PGC training–also known as sound-to-letter correspondence–for the target word being trained (Tsapkini and Hillis, 2013). Following the PGC training, participants must attempt to write the word corresponding to the picture a second time, with reinforcement provided upon successful completion. If unsuccessful, they are guided through the writing process: the speech-language therapist (or a trained research assistant), hereafter referred to as the therapist, shows the word again and provides PGC training while the participant copies it. In cases where participants improve substantially within the first sessions of intervention, the therapist encourages the participant to form sentences using the target words or engage in personally relevant conversations about the target word to ensure all participants receive at least 45 min of therapy.

As shown in Table 1, Figure 1, a therapist will administer therapy 5 days per week for three consecutive weeks. Therapy will last between 45 and 90 min, including the concomitant stimulation during the first 20 min. The exact duration of therapy will vary depending on the participant's tolerance for effort and speed of execution, and breaks will be taken if needed. Therapy will be conducted individually, in person, in a quiet room.

During each therapy session, the 20 words on the trained list (described in the next section) will be used for that period. To ensure therapy validity across different sites, a NaSp app was developed by the first author (MMT) in R using Shiny App. The app guides the therapist in following the NaSp flow according to the participant's answers. It allows the therapist to track the participant's spoken and written naming performance, as well as the effectiveness of cues when used. A comprehensive NaSp tutorial was also developed to help train therapists at the different sites.

##### Therapy stimuli

Two sets of therapy stimuli were developed for NaSp, targeting two levels of baseline performance. This structure reflects the clinical reality of variability in naming performance among individuals with PPA. It is guided by psycholinguistic evidence showing that lexical characteristics such as word frequency, length, imageability, and age of acquisition influence naming success (Vonk et al., 2019; Jebahi et al., 2023). This two-level approach offers a scalable and principled method for adapting therapy difficulty to participant ability, all while maintaining methodological consistency and control over psycholinguistic variables. Level 1 (easier words suitable for more moderate or advanced difficulties based on naming performance on the Level I list at the T1 assessment, < 70% correct) and Level 2 lists (more challenging words suitable for milder impairments based on naming performance on the Level 1 list at the T1 assessment, 70% or greater correct). Each level consists of 80 stimuli, comprising both nouns and verbs, and levels differ in psycholinguistic factors, based on the English Lexicon Project (Balota et al., 2007).

In Level 1, therapy words are characterized by shorter lengths [mean (*M*) ± standard deviation (*SD*)] 5.33 ± 1.23 letters for nouns and 5.63 ± 1.53 letters for verbs, higher frequency (21.20 ± 17.41 for nouns and 13.20 ± 21.29 for verbs), and an earlier age of acquisition (AoA; 5.15 ± 1.21 for nouns and 6.41 ± 1.68 for verbs). All words in this level are imageable, and corresponding pictures were sourced from the International Picture Naming Project (Székely et al., 2003; Szekely et al., 2005). Level 1 consists of a total of 40 nouns and 40 verbs.

Level 2 stimuli are longer (9.06 ± 1.02 letters for nouns and 9.31 ± 1.01 letters for verbs), of lower frequency (1.25± 1.29 for nouns and 0.75 ± 0.86 for verbs), and a later AoA (11.63 ± 2.25 for nouns and 11.07 ± 2.57 for verbs). Because not all words in this level are imageable, short definitions were crafted for all therapy stimuli to be read jointly with the presentation of the picture. Pictures for Level 2 were obtained from both the International Picture Naming Project (Székely et al., 2003; Szekely et al., 2005) and The Noun Project (2023). This level consists of 64 nouns and 16 verbs.^3^

Following the T1 assessment, the therapy level for each participant is determined based on their performance, and they remain at that level for all subsequent study periods. For each participant, four random lists are generated, each containing 20 items designated for treatment in Period 1, 20 for generalization measurement in Period 1, 20 for treatment in Period 2, and 20 for generalization measurement in Period 2. All lists are matched for the above-specified psycholinguistic factors, including AoA, frequency, and word length. Participants assigned to Level 2 are also probed on items from Level 1 at each assessment to help track general decline throughout the trial.

#### tDCS

**Figure 3:**
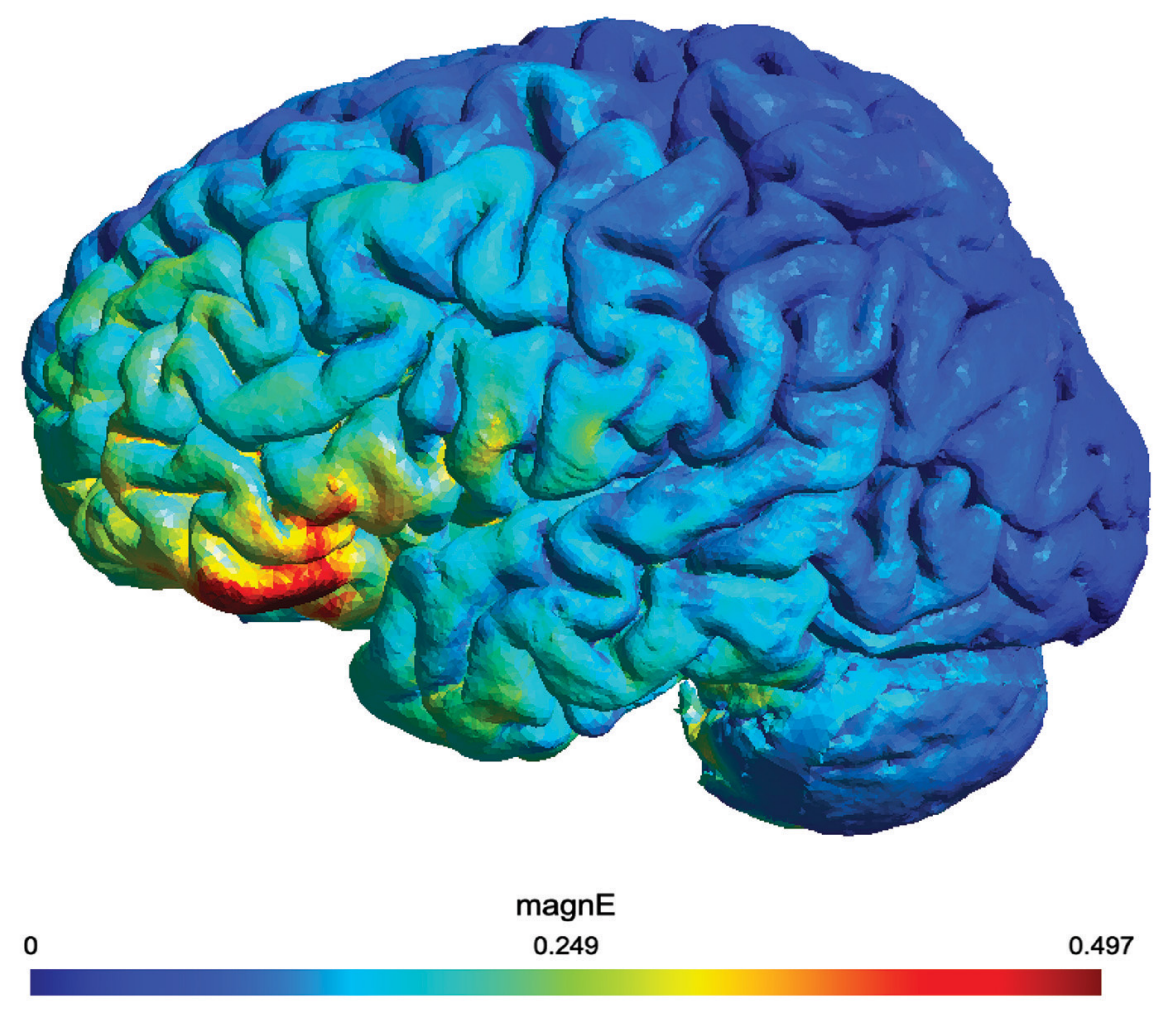
Electric field simulation of the tDCS montage created using SimNIBS 4.1.0 (Thielscher et al., 2015). The montage places the anode over the left inferior frontal gyrus (IFG) at the F7 location and the cathode extracephalically on the right cheek. The simulation visualizes current propagation and electric field intensity, with targeted stimulation of the left IFG. The color map represents the magnitude of the electric field (MagnE) in volts per meter (V/m).

The administration of tDCS involves using the Soterix 1x1 platform and 5 cm × 5 cm electrodes. The anode is positioned over the left frontal lobe at F7 according to the 10-20 electrode placement system, while the cathode is placed extracephalically over the right cheek. This unihemispheric tDCS montage aims to achieve more targeted stimulation, aligning with predictions from prior modeling studies (Moliadze et al., 2010; Wagner et al., 2007; Bhattacharjee et al., 2019). While the F7 position provides a standardized landmark for left IFG stimulation, it is important to acknowledge that individual anatomical differences such as cortical folding, sulcal depth, and skull thickness can influence the intensity and distribution of the electric field delivered to the target region (Laakso et al., 2015; Saturnino et al., 2019). However, modeling data in neurodegenerative populations suggests that such variability may not substantially compromise targeting, despite atrophy-related anatomical changes in individuals with PPA, consistent current flow patterns to target regions remained consistent across variants and individuals (Unal et al., 2020). While individual electric field modeling can improve focality, it remains resource-intensive and challenging to implement in large clinical trials. Moreover, in the context of neurodegenerative conditions like PPA, where functional reorganization and loss of network hubs may occur, increased focality may not always be advantageous (Mandelli et al., 2018; Agosta et al., 2014; Tao et al., 2020). In fact, when comparing conventional and high-definition tDCS, conventional tDCS seems to yield stronger effects, and it could be the lack of focality that make it very efficacious as it is discussed in Unal et al. (2020). These findings support the robustness of this tDCS montage in achieving reliable stimulation even in the presence of disease-related anatomical variability. See Figure 3 for the simulation of current propagation with the montage used in this study.

To minimize skin-electrode reactions, a non-metallic, conductive rubber electrode with a saline-soaked sponge is used to cover the left inferior frontal gyrus (IFG). Each tDCS session delivers 2 mA of current (with an estimated current density of 0.08 mA/cm^2^) for 20 min during the a-tDCS period, concurrent with the naming therapy, and is followed by at least 25 min of therapy alone or the time required to complete the trained list.

During sham stimulation (s-tDCS), a brief 30-s ramp-up and ramp-down of current mimic the sensation of active tDCS. This method has effectively blinded participants to the stimulation condition in prior studies (Palm et al., 2013). In earlier studies conducted by our team, including randomized, sham-controlled trials of tDCS in individuals with PPA, blinding procedures were shown to be effective. Specifically, post-trial assessments of participant and care partner guesses regarding stimulation condition (active vs. sham) revealed accuracy rates at chance (Tsapkini et al., 2018) or were unable to identify a difference (Roncero et al., 2019), suggesting successful blinding in this population.

To monitor adverse effects, each participant is asked to rate their pain level once at the end of each stimulation session using the Wong-Baker FACES Pain Rating Scales (http://www.WongBakerFACES.org) (Wong-Baker FACES Foundation, 2022). The protocol requires each participant to undergo 15 consecutive weekday sessions.

After completing the trial, at T7, participants will also be asked to indicate during which period they believe they received active or sham stimulation (using a dichotomous response format). This protocol ensures comprehensive data collection while maintaining participant safety and minimizing potential sources of bias.

### Outcome measures

Each site has designated assessors trained to use the materials developed for this RCT to standardize assessments across sites. These materials include an assessment manual, a dynamic Microsoft PowerPoint presentation (referred to as the *Assessment Presentation*), and a scoring sheet booklet. As shown in Table 1, Figure 1, assessments are repeated seven times over a period of 33 weeks. All assessment tasks are organized in these materials in the order of administration and divided into four balanced working blocks. The assessments can be completed over two full days or across four half-days within a week.

All participant instructions are integrated into the Assessment Presentation and appear on the scoring sheets, along with additional administration details as necessary. The timing constraints are embedded in the presentation for tasks with time limits to ensure consistent administration throughout each site involved. The assessment manual includes further scoring guidelines, task-specific details, and an FAQ section for each task. Inter-site meetings are organized as needed to discuss scoring questions, and the decisions are documented in the assessment manual.

Finally, all assessment data will be collected and managed using REDCap electronic data capture tools hosted at Johns Hopkins University (Harris P. A. et al., 2019; Harris et al., 2009). REDCap (Research Electronic Data Capture) is a secure, web-based software platform designed to support data capture for research studies, providing (1) an intuitive interface for validated data capture; (2) audit trails for tracking data manipulation and export procedures; (3) automated export procedures for seamless data downloads to standard statistical packages; and (4) procedures for data integration and interoperability with external sources.

#### Primary outcome measures

The primary outcome measure will be the change in performance between time points, as measured by phonemic accuracy (during spoken naming) and letter accuracy (during spelling) of trained items. Phonemic and letter accuracy will be calculated on a scale of 0–100%, with a higher number reflecting higher accuracy, using the Phonology analysis and Spelling analysis tools in Open Brain AI (Themistocleous, 2023).

At each assessment time point, participants undergo a comprehensive evaluation that includes the NaSp spoken naming and spelling task, which serves as the primary outcome measure. The task consists of 80 stimuli corresponding to the Level 1 stimuli previously outlined in the Therapy Stimuli section (along with 80 items included in Level 2 for identified participants). Accuracy on trained items is then computed separately.

The assessor presents all the words in the list. For each noun, participants attempt to name the item orally and then write their answer. No feedback is given, as some items serve as generalization items for either P1 or P2. After assessing the nouns, the same procedure is applied to the verbs.

All spoken and written responses are recorded. Spoken responses will be transcribed manually using the International Phonetic Alphabet, and written responses are captured using a smart pen (Symphony Smartpen by Livescribe, n.d.) to ensure accurate data collection. Both the spoken and written responses will be typed into a scoring document.

#### Secondary outcome measures

The secondary outcome measures will be the changes in performance between time points, specifically phonemic accuracy (during spoken naming) and letter accuracy (during spelling) of untrained items. Similar to the primary outcome measure, phonemic and letter accuracy will be calculated on a scale of 0–100%, with higher numbers indicating greater accuracy, using the Phonology analysis and Spelling analysis tools in Open Brain AI (Themistocleous, 2023). The performance on the NaSp spoken naming and spelling task will be used to compute the accuracy of the untrained items.

Additionally, resting-state functional MRI (rs-fMRI) will be used to measure the activity of various brain regions during resting or task-negative conditions. This approach will allow for the evaluation of functional interactions between brain regions. Further details regarding the neuroimaging methods are provided below.

#### Other pre-specified outcome measures

##### Neurocognitive tests

Other pre-specified outcome measures in this trial include neurocognitive tests for broader language and cognitive domains, as well as standardized questionnaires for patient-reported outcome measures (PROM) covering communication and functional abilities.

In particular, we will compute changes in:

##### Language outcomes (for each type of task, corresponding tests are listed):

- Oral naming tasks
  ° Boston Naming Test (BNT) short version (Goodglass and Kaplan, 1983; Mack et al., 1992): a measure of spoken naming abilities for nouns. It provides a total score (score range: min = 0, max = 30; higher score = better outcome) and a total score after phonemic cueing (score range: min = 0, max = 30; higher score = better outcome).
  ° Philadelphia Naming Test-short form A (PNT) (Walker and Schwartz, 2012): a measure of spoken naming abilities for nouns. The test contains 1- and 2-syllable words, as well as low, medium, and high-frequency words. It provides a total score (score range: min = 0, max = 30; higher score = better outcome).
  ° Hopkins Assessment of Action Naming (HANA) (Breining et al., 2022): a measure of spoken naming abilities for verbs. It provides a total score (score range: min = 0, max = 30; higher score = better outcome).
  ° Verb and Sentence Test (VAST) Action Naming (Bastiaanse et al., 2003): a measure of spoken naming abilities for verbs. The test contains transitive and intransitive verbs and low- and high-frequency verbs. It provides a total score (score range: min = 0, max = 40; higher score = better outcome), a high-frequency score (score range: min = 0, max = 19; higher score = better outcome), a low-frequency score (score range: min = 0, max = 21; higher score = better outcome), a transitive verb score (score range: min = 0, max = 29; higher score = better outcome), an intransitive verb score (score range: min = 0, max = 11; higher score = better outcome), a name-related verb score (score range: min = 0, max = 18; higher score = better outcome) and a non-name-related transitive verb score (score range: min = 0, max = 22; higher score = better outcome).
- Spelling to Dictation
  ° Johns Hopkins University (JHU) Dysgraphia Battery using the Oral Spelling list (Goodman and Caramazza, 1985): a measure of spelling to dictation abilities. This test contains words and non-words. It provides a total score (score range: min = 0, max = 62; higher score = better outcome), a word score (score range: min = 0, max = 43; higher score = better outcome), a non-word score (score range: min = 0, max = 19; higher score = better outcome) and a letter accuracy score (score range: min = 0, max = 100%; higher score = better outcome).
  ° In-house Spelling to Dictation list: a measure of spelling to dictation abilities. This test contains words from a range of imageability and frequency. It provides a total score (score range: min = 0, max = 30; higher score = better outcome) and a letter accuracy score (score range: min = 0, max = 100%; higher score = better outcome).
- Morphosyntax Production
  ° Verb and Sentence Test (VAST) Filling in Finite Sentences (Bastiaanse et al., 2003): a measure of verb retrieval in a finite sentence context. The test contains transitive and intransitive verbs and low- and high-frequency verbs. It provides a total score (score range: min = 0, max = 10; higher score = better outcome).
  ° Verb and Sentence Test (VAST) Filling in Infinite Verbs in Sentences (Bastiaanse et al., 2003): a measure of verb retrieval in an infinite sentence context. The test contains transitive and intransitive verbs and low- and high-frequency verbs. It provides a total score (score range: min = 0, max = 10; higher score = better outcome).
  ° Verb and Sentence Test (VAST) Sentence Construction (Bastiaanse et al., 2003): measures sentence production abilities. The test contains transitive and intransitive verbs and reversible and irreversible verbs. It provides a total score (score range: min = 0, max = 20; higher score = better outcome), an intransitive verb score (score range: min = 0, max = 10; higher score = better outcome), a transitive verb score (score range: min = 0, max = 10; higher score = better outcome), a reversible transitive verb score (score range: min = 0, max = 5; higher score = better outcome), an irreversible transitive verb score (score range: min = 0, max = 5; higher score = better outcome).
- Sentence Comprehension
  ° SOAP sentence comprehension (Love and Oster, 2002): measures sentence comprehension abilities. This task contains four syntactic construction types (matched for length): active, passive, subject-relative, and object-relative, either at the active or passive tense. It provides an active score (score range: min = 0, max = 10; higher score = better outcome), a passive score (score range: min = 0, max = 10; higher score = better outcome), a subject relative score (score range: min = 0, max = 10; higher score = better outcome) and an object relative score (score range: min = 0, max = 10; higher score = better outcome).
- Repetition
  ° Frontotemporal Lobar Degeneration Module (FTLD-MOD) sentence repetition task—extended (Ruch et al., 2022): a measure of repetition abilities. It provides a total score for the original sentences (score range: min = 0, max = 5; higher score = better outcome), a number of omitted words for the original sentences score (score range: min = 0, max= 37, higher score = worse outcome), a number of semantically related or unrelated incorrect real words score for the original sentences (score range: min = 0, max= 37, higher score = worse outcome), a number of phonologically related words or non-words score for the original sentences (score range: min = 0, max= 37, higher score = worse outcome), a total score for the new sentences (score range: min = 0, max = 5; higher score = better outcome), a number of omitted words for the new sentences score (score range: min = 0, max= 63, higher score = worse outcome), a number of semantically related or unrelated incorrect real words score for the new sentences (score range: min = 0, max= 63, higher score = worse outcome), a number of phonologically related words or non-words score for the new sentences (score range: min = 0, max= 63, higher score = worse outcome).
- Verbal fluency
  ° Letter criteria [letter (f, a, and s)]: a measure of verbal fluency abilities (score range min = 0, max = no limits; higher score = better outcome).
  ° Semantic criteria (animals, fruits, and vegetables): a measure of verbal fluency abilities (score range min = 0, max = no limits; higher score = better outcome).
- Connected speech:
  ° Boston Diagnostic Aphasia Examination (BDAE) Cookie Theft (Goodglass and Kaplan, 1983; Goodglass et al., 2001): a measure of oral production. It provides a Correct Information Units (CIU) score (score range: min = 0, max = 8; higher score = better outcome), a total number of words score (score range: min = 0, max = no limits; higher score = better outcome), a ratio of nouns/total number of words (score range: min = 0, max = 1; higher score = better outcome), a ratio of verbs/total number of words (score range: min = 0, max = 1; higher score = better outcome), a ratio of subordinates/number of sentences (score range: min = 0, max = no limits; higher score = better outcome), a ratio of phonological errors/number of words (score range: min = 0, max = 1; higher score = worse outcome), and a ratio of lexico-semantic errors/number of words (score range: min = 0, max = 1; higher score = worse outcome).
  ° Apraxia Battery for Adults (ABA) Circus Scene (Dabul, 2000): a measure of oral production. It provides a CIU score (score range: min = 0, max = 8; higher score = better outcome), a total number of words score (score range: min = 0, max = no limits; higher score = better outcome), a ratio of nouns/total number of words (score range: min = 0, max = 1; higher score = better outcome), a ratio of verbs/total number of words (score range: min = 0, max = 1; higher score = better outcome), a ratio of subordinates/number of sentences (score range: min = 0, max = no limits; higher score = better outcome), a ratio of phonological errors/number of words (score range: min = 0, max = 1; higher score = worse outcome), and a ratio of lexico-semantic errors/number of words (score range: min = 0, max = 1; higher score = worse outcome).
  ° Disease and life events' prompts (Nicholas and Brookshire, 1995): a measure of oral production. It provides a CIU score (score range: min = 0, max = 8; higher score = better outcome), a total number of words score (score range: min = 0, max = no limits; higher score = better outcome), a ratio of nouns/total number of words (score range: min = 0, max = 1; higher score = better outcome), a ratio of verbs/total number of words (score range: min = 0, max = 1; higher score = better outcome), a ratio of subordinates/number of sentences (score range: min = 0, max = no limits; higher score = better outcome), a ratio of phonological errors/number of words (score range: min = 0, max = 1; higher score = worse outcome), and a ratio of lexico-semantic errors/number of words (score range: min = 0, max = 1; higher score = worse outcome).

##### Executive functions and learning

- Rey Auditory Verbal Learning Test (RAVLT) (Rey, 1941): a measure of list learning (immediate recall) and long-term memory (delayed recall). It provides a Total Recall score (the number of correct words learned over five trials, score range: min = 0, max = 75, higher score = better outcome), a Delayed Recall score (the number of correct words recalled after a 20-min delay, score range: min = 0, max = 15, higher score = better outcome), Recognition score (the score calculated by subtracting false positives from recognition, score range: min = 0, max = 15, higher score = worse outcome).
- Digit spans forward and backward—task from the Wechsler Adult Intelligence Scale—revised (WAIS-R) (Wechsler, 1981): a measure of short-term memory and working memory (separate score for forward and backward, the length of the maximum span correctly repeated, score range: min = 1.0, max = 7.0, higher score = better outcome).
- Spatial spans forward and backward (Corsi, 1972): a measure of non-verbal short-term memory and working memory (separate score for forward and backward, the length of the maximum span correctly answered, score range: min = 1.0, max = 7.0, higher score = better outcome).
- Ravens Colored Progressive Matrices (RCPM) (Raven and Raven, 2003): a measure of problem-solving skills, logical reasoning, attention, and ability to learn (the total number of correct answers, score range: min = 0, max = 36; higher score = better outcome).
- Trail Making Test (TMT) (Stuss et al., 2001): a measure of attentional abilities. It provides a total score in time (score range: min = n/a, max = 300 s; higher score = worse outcome), a Part A score in time (score range: min = n/a, max = 300 s; higher score = worse outcome), a Part B score in time (score range: min = n/a, max = 300 s; higher score = worse outcome), a difference score (score range: min = n/a, max = n/a; higher score = worse outcome) and a ratio score (score range: min = n/a, max = n/a; higher score = worse outcome).
- Attention—Attention Network Test (ANT) (Fan et al., 2005): a measure of the effects of cues and targets within a single reaction time task. The task provides RTs for congruent and incongruent trials, either cued or non-cued. Three scores are derived from these trials: orienting, alerting and detection, all involved in attention. These scores reflect different components of executive functions (score range: min = n/a, max = n/a; higher score = worse outcome).

##### Lifestyle questionnaires

- Caregiver Burden Questionnaire (Zarit et al., 1980): questionnaire filled only by the caregiver as a measure of the degree of burden, made with a 29-item self-report inventory (score range: min = 0, max = 84; higher score = worse outcome).
- Communication Aphasia Test (CAT): Disability Questionnaire (Swinburn et al., 2012): questionnaire filled by both the individual with PPA and their caregiver as a measure of communication difficulties. Questions cover reading, writing, speaking, and understanding (score range: min = 0, max = 64; higher score = worse outcome).
- Communication Confidence Rating Scale for Aphasia (CCRSA) (Cherney et al., 2011): questionnaire made of 10 questions to answer with a percentage scale (from 0% to 100%) filled by both the individual with PPA and their caregiver as a measure on communication confidence (score range: min = 0, max = 100; higher score = better outcome).
- Clinical Dementia Rating Scale (CDR) (Morris, 1993): questionnaire filled only by the caregiver as a measure of dementia severity and progression in FTD (score range: min = 0, max = 24; higher score = worse outcome).
- Dementia Quality of Life Scale (DEM-QOL) © Institute of Psychiatry King's College of London (Smith et al., 2005): questionnaire filled by both the individual with PPA and their caregiver (score range: min = 28, max = 112; higher score = better outcome).
- Exercise Questionnaire (in-house questionnaire developed by Ficek, B.) : qualitative questionnaire filled by both the individual with PPA and their caregiver describing the individual with PPA's level of weekly physical activity.
- Instrumental Activities of Daily Living (IADL) (Lawton and Brody, 1969): questionnaire filled out only by the caregiver (score range: min = 0, max = 8, higher score = better outcome)
- Language Experience and Proficiency Questionnaire (LEAP-Q) (Marian et al., 2007): questionnaire filled only by the caregiver to rate the individual with PPA's proficiency in other languages using an 11-point Likert scale (0–10).
- Progressive Aphasia Severity Scale Questionnaire (PASS) (Sapolsky et al., 2014): a measure of the severity and progression of language deficits in patients with PPA filled by the caregiver. The scale evaluates 13 linguistic domains (each with scores ranging from 0 to 3, higher score = worse outcome). These domains include articulation, fluency, syntax and grammar, word retrieval and expression, repetition, auditory comprehension, single-word comprehension, reading, writing, functional communication, conversation, turn-taking during conversation, and language generation.
- Patient Health Questionnaire (PHQ-9) (Kroenke et al., 2001): questionnaire filled by both the individual with PPA and their caregiver (score range: min = 0, max = 27; higher score = worse outcome).
- Pittsburgh Sleep Quality Index (PSQI) (Buysse et al., 1989): consists of 19 self-rated questions and five questions rated by the bed partner or caregiver, grouped into seven component scores: subjective sleep quality, sleep latency, sleep duration, habitual sleep efficiency, sleep disturbances, use of sleeping medications, and daytime dysfunction (score range for each component: min = 0, max = 21; higher score = worse outcome).
- Resilience Evaluation Scale (RES) (van der Meer et al., 2018): questionnaire with eight questions filled by both the individual with PPA and their caregiver (score range: min = 0, max = 36; higher score = better outcome).
- Survey of Autobiographical Memory (SAM) © Baycrest, 2012 (Palombo et al., 2013): questionnaire made of 26 items to answer using a 5-point Likert scale (1: completely disagree to 5: completely agree) covering four memory domains (episodic, future, semantic, and spatial) filled by both the individual with PPA and their caregiver (episodic score range: min = 0, max = 45; higher score = worse outcome; future score range: min = 0, max = 30; higher score = worse outcome, semantic score range: min = 0, max = 30; higher score = worse outcome, spatial score range: min = 0, max = 30; higher score = worse outcome).

Surrogate outcome measures include several MRI sequences, genetic sequencing, and biomarkers. As shown in Table 1 and Figure 1, MRI exams and biomarker samplings are performed at T1, T4, and T7.

#### Neuroimaging

MRI protocols for structural and resting-state functional MRI will follow those established by the Alzheimer's Disease Neuroimaging Initiative (ADNI). This large, multi-center, and multi-manufacturer study offers standardized protocol implementations that can be harmonized across different MRI devices, ensuring consistency and comparability of imaging data across various research sites. Each participating site will adapt the ADNI-3 protocol to their specific scanner hardware and software while maintaining core acquisition parameters to ensure harmonization. Structural and functional connectivity will be measured using the following methods based on the ADNI-3 protocol (representative parameters):

- T1-weighted structural MRI (MPRAGE): Used for volumetric and cortical thickness analysis. ADNI-3 representative parameters: TR = 8.8 ms, TE = 1.9 ms, flip angle = 20°, FOV = 256 × 256 mm^2^, voxel size = 1 × 1 × 1 mm3, 176 slices, no gap.
- T2-weighted structural MRI: Fast spin echo (FSE) sequence for visualization of white matter lesions and anatomical delineation. ADNI-3 representative parameters: TR = 3,000–3,700 ms, TE = 80–100 ms, voxel size = 1 × 1 × 1 mm3, 176 slices.
- FLAIR imaging: Included to assess white matter hyperintensities/microvascular lesions, as recommended by ADNI-3.
- Diffusion tensor imaging (DTI): Used for structural connectivity analysis. ADNI-3 representative protocol: 232 × 232 × 160 mm at 2 × 2 × 2 mm, TE = 71 TR=3,300, three shells (b = 500,1,000, 2,000 s/mm^2^), 112 total diffusion-weighted directions, scan time ≈ 7 min 10 s.
- Resting-state functional MRI (RS-fMRI): Used for functional connectivity analysis. ADNI-3 representative sequence: 2D EPI sequence with SENSE acceleration, in-plane resolution = 3.3 × 3.3 mm^2^, 64 × 64 voxels; TR = 2,500 ms, TE = 30 ms, flip angle = 75°; slice thickness = 3 mm; 250 time points, scan time ≈ 11 min.

##### Neuroimaging analysis

###### Gray/white matter ratio

Using SPM12 (Wellcome Department of Imaging Neuroscience Group, 2014) Tissue Volume utility (Malone et al., 2015), we will segment MPRAGE images into gray and white matter and calculate each subject's gray/white ratio within the region of interest (ROI) beneath the tDCS site. This involves adjusting for volume changes and correcting for intensity non-uniformities.

###### Region-of-interest (ROI) volumetric analysis

ROI volumetric analysis following the automated segmentation of the MPRAGE using MRI Cloud (Mori et al., 2016).

###### Cortical thickness

FreeSurfer software (Fischl, 2012) will measure cortical thickness within the ROI underlying the tDCS site. The Advanced Normalization Tools software (ANTs) (Avants et al., 2009) will map MNI volumetric coordinates to FreeSurfer's surface-based coordinate system.

###### Total intracranial volume (ICV)

ICV will be computed for each subject using SPM12 (Wellcome Department of Imaging Neuroscience Group, 2014) Tissue Volume utility (Malone et al., 2015) to measure the volumes of gray matter, white matter, and CSF, which are then summed to calculate ICV. This helps account for potential differences in ICV between men and women and its potential impact on tDCS effectiveness.

###### DTI image analysis

MRICloud (Mori et al., 2016) will automatically preprocess and segment diffusion-weighted images, focusing on DTI scalars such as fractional anisotropy and mean diffusivity, and segment them into 169 regions of interest. We will concentrate on areas of the language network underlying the tDCS site.

###### RS-fMRI analysis

MRICloud (Mori et al., 2016) will preprocess resting-state fMRI scans, coregistering them with MPRAGE scans, and parcel them into 283 segments using a multi-atlas fusion label algorithm. Standard preprocessing includes corrections for motion and physiological noise, and average time courses are extracted and normalized for 78 ROIs. Correlations between region pairs are calculated and transformed using the Fisher z-transformation.

#### Genetic factors

Saliva samples will be collected for processing to extract DNA. Saliva samples will be stored at room temperature and processed in batches.

- Saliva samples will be collected using the OG-100 Oragene collection kit. DNA will be extracted using standard methods, and genotyping will be performed at the Johns Hopkins DNA Diagnostic Laboratory utilizing Taqman assays and an ABI7900HT apparatus.

##### Genetic sequencing

DNA extracted from saliva samples will be analyzed to identify specific gene polymorphisms that may influence neuroplasticity and cognitive function. The gene polymorphisms of interest include:

- Brain-derived neurotrophic factor (BDNF) gene: The Val66Met polymorphism, resulting from a G/A substitution at codon 66, leads to either a valine (Val) or methionine (Met) substitution. Individuals may carry one of three variations: Val/Val, Val/Met, or Met/Met.
- Catechol-o-methyl transferase (COMT) gene: The Val158Met polymorphism, involving a G/A substitution at codon 158, results in Val/Val, Val/Met, or Met/Met variations.
- Apolipoprotein E (ApoE) gene: Variants of the ApoE gene include ε2, ε3, and ε4 alleles, with possible combinations such as ε3/ε3, ε3/ε4, or ε4/ε4.

#### Biomarkers

Blood samples will be collected and processed to extract relevant biomarkers.

- Blood will be drawn from veins in the forearm using a standardized protocol. Blood is collected into two pre-chilled lavender-top EDTA tubes and one room-temperature red-top serum tube.
- Samples are inverted immediately after collection and stored on ice or at room temperature as appropriate. Processing and freezing are completed within 60 min of collection.

##### Biomarker analysis

The following biomarkers will be analyzed in the blood sample to measure the concentration of each:

- Neurofilament Light Chain (NfL)
- Serum Tau
- Amyloid-β (Aβ)

### Statistical analysis

This trial investigates whether combining tDCS targeting the left IFG with a tailored spoken naming and spelling therapy improves language abilities and communication in individuals with PPA, with expected benefits extending to untrained tasks and lasting up to 3 months. It also aims to identify predictors of treatment response.

The primary outcomes will be analyzed using a general crossover formulation that considers each follow-up time point, the effects of treatment (s-tDCS vs. a-tDCS), phase (P1 or P2), and their interaction. For each participant, the data will include the order of treatments (s-tDCS in phase 1 and a-tDCS in phase 2, or vice versa) and changes in accuracy from before to after each phase for phonemic accuracy (during spoken naming) and letter accuracy (during spelling) of trained items.

The general crossover analysis will estimate parameters such as intervention effects in each phase and the overall average intervention effect. Estimates of these effects, standard errors, and confidence intervals will be calculated using the generalized estimating equation (GEE) method with robust variance estimation to account for correlations among repeated outcomes across different time points and phases within an individual. *P*-values will be exact and derived by comparing the estimates to the distribution calculated by permuting the order of intervention assignment across patients.

To address potential carryover effects inherent in the within-subject crossover design, a period-by-treatment interaction term will be included in the GEE model. A significant interaction will indicate possible carryover. In this case, follow-up sensitivity analyses will be conducted such as restricting analyses to Period 1 only, to confirm whether the treatment effect is robust. Washout adequacy will also be examined based on baseline performance at each phase.

To identify the predictors of differential effects of tDCS vs. sham on primary outcomes, we will separately analyze clinical (PPA variant, severity of impairments), neural (functional and structural), genetic, cognitive, and demographic (age, sex) factors, as well as lifelong language experience (e.g., bilingualism) factors. We will employ a model comparison approach, evaluating each predictor type based on effect size and cross-validated R-squared variance. By sequentially adding each predictor type, we will measure the variance contributed by each factor and establish a threshold for acceptable variance from each factor. To account for multiple comparisons across the range of predictors tested, we will apply False Discovery Rate (FDR) correction using the Benjamini-Hochberg procedure (Benjamini and Hochberg, 1995). This approach controls the expected proportion of false positives among the predictors identified as significant, ensuring a balanced trade-off between Type I error control and discovery potential in the context of exploratory modeling.

Using cross-validated, R-squared model selection minimizes the need for extensive adjustments for multiple comparisons. We will also assess the significance of the remaining factors by comparing them to the bootstrap distribution of sorted t-statistics under the null hypothesis that no factors predict the tDCS vs. sham effect. This final model will help us identify individuals who demonstrate a statistically significant difference between the effects of tDCS and sham treatment.

## Discussion

The treatment of language difficulties in PPA remains a significant challenge and a high-priority, unmet medical need. Addressing this complex issue requires a transformative approach. Recent advances in communication disorder treatments for PPA have largely come from non-invasive neuromodulation techniques, such as tDCS. This RCT stands to be the first multisite Phase II study evaluating tDCS's efficacy as an adjuvant for behavioral interventions in PPA therapy, representing a critical step toward addressing this need.

This trial employs a randomized, double-blind, sham-controlled, within-subject crossover design, with follow-ups conducted immediately after therapy and at 4 weeks and 3 months post-therapy. It evaluates the effectiveness of tDCS administered over left perisylvian language areas, combined with a novel, cognitive model-driven behavioral therapy called NaSp. Together, these interventions aim to enhance spoken naming and spelling abilities and ultimately communication in individuals living with lvPPA and nfvPPA. Intending to replicate and validate prior findings, this study seeks to advance evidence on combining neuromodulation techniques with established language interventions.

This study population consists of individuals living with lvPPA or nfvPPA. Individuals living with svPPA are intentionally excluded, which we acknowledge limits the generalizability of the findings. However, this decision was based on both theoretical and empirical considerations. svPPA is characterized by profound semantic deficits, resulting in degraded word meaning and impaired object knowledge, which distinguish it from the phonological and graphemic impairments seen in lvPPA and nfvPPA (Gorno-Tempini et al., 2011; Sepelyak et al., 2011). These semantic deficits have been shown to reduce responsiveness to therapies targeting lexical retrieval and limit the generalization to items beyond those that have been trained. In prior work, individuals with svPPA demonstrated learning for trained items but failed to generalize the gains (Tsapkini et al., 2018; Roncero et al., 2019), a finding echoed in recent reviews (Norata et al., 2023). Individuals living with svPPA have been shown to respond better to interventions focusing on semantic knowledge and “look, listen, repeat” methods (Suarez-Gonzalez et al., 2021), or following stimulation of temporal areas (Teichmann et al., 2016), which is not the focus of this trial. By focusing on lvPPA and nfvPPA, the present trial targets populations with stronger evidence of responsiveness and generalization, enabling a more rigorous evaluation of tDCS as an adjuvant to lexical retrieval therapy.

The NaSp therapy protocol itself is an adaptation of previous work, refined specifically to target spoken naming and address prior limitations as the previous study focused on spelling. This updated protocol enhances the potential therapeutic effect for individuals with PPA, especially through its adaptability. Specifically, NaSp enables the tailoring of task difficulty to meet the needs of each participant, aligning with the principles of precision medicine. However, a limitation in the creation of the therapy lists is that trained and untrained items are not individualized; baseline performance does not influence the selection process. This lack of item-specific customization could, in some cases, obscure the therapy's effect, as the trained list may predominantly include words the participant already finds relatively easy, thereby minimizing measurable gains.

To optimize customization, we introduce two training lists: Level 1, which contains more frequent, shorter words, and Level 2, comprising less frequent, longer, and sometimes more abstract words. Recognizing that some Level 2 words are not readily imageable, we included definitions alongside images to provide clearer context. While the Level 2 stimuli may have limitations, they were incorporated to offer valuable intervention options, especially for participants with recent diagnoses and milder impairments. This decision reflects our commitment to providing meaningful therapeutic opportunities to all participants, regardless of their impairment severity, and to avoid the typical scenario of leaving individuals with milder PPA symptoms without intervention options. Moreover, for participants who progress quickly through these stimuli, an additional component was introduced: the option to contextualize target words, enhancing semantic cueing and expanding to sentence-level stimulation. This added layer aims to strengthen the semantic aspect of the intervention, further enriching the therapeutic experience and potentially improving long-term outcomes.

If this trial does not show a clear benefit of a-tDCS over sham stimulation, several explanations would need to be considered. One possibility is that tDCS, as administered in this protocol (i.e., targeting the left IFG with 2 mA of current for 20 min), does not meaningfully enhance language therapy outcomes in individuals with lvPPA and nfvPPA. However, a lack of effect may also point to methodological factors such as individual variability in anatomy, neurodegeneration severity, or stimulation response that mask potential benefits. Alternatively, the behavioral protocol itself, although based on previously validated work, may not optimally interact with neuromodulation in all participants. Null findings could thus guide refinements in personalization strategies, including dose-response optimization, better stratification by neurobiological or genetic markers, and further integration of imaging data to predict responsiveness. Importantly, even in the absence of a group-level benefit, this trial's rich dataset, including behavioral, imaging, and biomarker measures, can still reveal important predictors of response and inform future precision-medicine approaches.

Finally, implementing this RCT has not been without its challenges. The multicentric nature of the study introduced several hurdles, including the involvement of diverse personnel with varying work habits and levels of familiarity with different tests, as well as significant physical distances between sites. These challenges compelled us to devise solutions that ultimately strengthened the project. As a result, we developed the Assessment Manual, which contains sections to document all decisions regarding scoring and a standard operating procedure (SOP) for assessments. Additionally, we created the NaSp app to facilitate the administration of the therapy, a comprehensive NaSp tutorial, and the NaSp fidelity checklist. These resources enhanced the project's robustness, necessitating careful planning and consideration from the outset. Meetings are also scheduled to address administrative questions and scoring issues, further ensuring the integrity of the study.

## Conclusions

This trial evaluates the efficacy of tDCS targeting the left IFG, combined with NaSp therapy, in enhancing naming and spelling in individuals living with PPA. Previous single-site studies including the Phase II focusing on spelling (Tsapkini et al., 2018) have shown that tDCS can augment language therapy outcomes, but there were no Phase II multisite trial has yet tested this approach.

This trial innovatively applies a precision medicine framework to tailor the difficulty of NaSp therapy to individual linguistic needs, while assessing critical predictors of treatment response, including genetic, neurofunctional, and cognitive variables. By integrating these domains, the study aims to advance our understanding of the individual factors that influence responses to therapy. This could lay the groundwork for more tailored interventions in the future.

Should the trial demonstrate positive outcomes, findings could inform clinical decision-making and support future implementation of scalable, targeted interventions. Future research may evaluate the feasibility of implementing tDCS in rehabilitation settings, explore long-term maintenance effects in real-world contexts, expand on recent work in home-based tDCS (Neophytou et al., 2024), and investigate variant-specific protocols (for example, adapting stimulation parameters and behavioral therapy to the needs of individuals living with svPPA). Ultimately, this research could help clinicians identify individuals most likely to benefit from combined tDCS and behavioral therapy, thereby enhancing care strategies for this complex and underserved demographic.

## Footnotes

^1^Restitutive therapy is used in this paper, and considered a synonym to remedial therapy, restorative treatment and impairment-based therapy.

^2^By daily life participation, we mean involvement in life situations, and more particularly the ability to engage in meaningful communication activities and social interactions with familiar and unfamiliar communication partners.

^3^It proved very challenging to find 40 verbs that met the targeted psycholinguistic parameters as verbs are more frequent than nouns in English (Kuperman and Van Dyke, 2013).
