# Supplementary material for: Protocol for a multisite study on the efficacy of transcranial direct current stimulation as an adjuvant to naming and spelling therapy in the treatment of oral and written naming in individuals with primary progressive aphasia
Source: Front Hum Neurosci. 2025 Sep 2;19:1611272. doi: 10.3389/fnhum.2025.1611272 (PMC12409816; doi:10.3389/fnhum.2025.1611272)
Supplement: Supplementary file 1 [file Table_1.docx]

Supplementary Material

# Supplementary Figures and Tables

Table 1. Standard Protocol Items: Recommendations for Interventional Trials (SPIRIT) details for the schedule of enrollment, interventions, and assessments, detailed version.

|  |  | STUDY PERIOD | | | | | | |
| --- | --- | --- | --- | --- | --- | --- | --- | --- |
|  | Enrollment | Post-allocation | | | | | | Close-out |
| TIMEPOINT | t_0_ | t_1_ | t_2_ | t_3_ | t_4_ | t_5_ | t_6_ | t_7_ |
| *ENROLLMENT* | | | | | | | | |
| Eligibility screen | X |  |  |  |  |  |  |  |
| Informed consent | X |  |  |  |  |  |  |  |
| Obtaining medical record | X |  |  |  |  |  |  |  |
| Allocation to research arm |  | X |  |  |  |  |  |  |
| *INTERVENTIONS* | | | | | | | | |
| Intervention period 1  (NaSp + a-tDCS or s-tDCS) |  |  |  |  |  |  |  |  |
| Intervention period 2  (NaSp + s-tDCS or a-tDCS) |  |  |  |  |  |  |  |  |
| *ASSESSMENTS* | | | | | | | | |
| **Baseline measures**  MoCA  CDR  Behavior-Comportment-Personality and Language Domains of the FTD-CDR^3^ |  | X |  |  |  |  |  |  |
| **Primary outcomes**  Trained items list  **Secondary outcomes**  Untrained items list  *MRI scan*  RS-fMRI |  | X | X | X | X | X | X | X |
| **Other pre-specified outcomes**  *Language*  Oral picture naming (nouns and verbs) BNT PNT HANA VAST Action Naming  Spelling to dictation  JHU Dysgraphia Battery (Oral Spelling list) In-house Spelling to Dictation  Morphosyntax production  VAST Filling in Finite Sentences VAST Filling in Infinite Verbs in Sentences VAST Sentence Construction  Sentence comprehension SOAP sentence comprehension  Repetition FTLD-MOD sentence repetition task  Connected speech BDAE Cookie Theft ABA Circus Scene Disease and life events prompts  Verbal fluency  Letter (f, a, and s)  Semantic (animals, fruits, and vegetables))  *Executive functions and learning*  RAVLT  Digit spans forward and backward  Spatial spans forward and backward  RCPM  TMT  Global Local  Simon task  Detection  ANT  N-back (1-back, 2-back, 3-back)  *Lifestyle questionnaires*  Caregiver Burden Questionnaire **  CAT Disability Questionnaire *  CCRSA *  CDR **  DEM-QOL *  Exercise Questionnaire *  FTLD-CDR ^**^  IADL **  LEAP-Q **  PASS **  PHQ-9 *  PSQI *  RES *  SAM * |  | X | X | X | X | X | X | X |
| *Other measures*  FACES scale  Blinding questionnaire |  |  |  |  |  |  |  | X |
| **Surrogate outcome markers**  *MRI scans*  MPRAGE  DTI |  | X |  |  | X |  |  | X |
| **Biomolecular data**  *Saliva sample*  BDNF  COMT  APOE4  *Blood sample*  NfL  Serum Tau  A-β |  | X  X |  |  | X |  |  | X |
| Interventions: NaSp: Naming and Spelling therapy, a-tDCS: active-transcranial direct current stimulation, s-tDCS: sham-transcranial direct current stimulation  Assessments: Amyloid-beta (A-β), Apraxia Battery for Adults (ABA) (Dabul, 2000), Attention Network Test (ANT) (Fan et al., 2005), Behavior-Comportment-Personality and Language Domains of the Frontotemporal Lobar Degeneration-Modified Clinical Dementia Rating (FTLD-CDR) (Knopman et al., 2008), Boston Diagnostic Aphasia Examination (BDAE) (Goodglass & Kaplan, 1983), Brain-Derived Neurotrophic Factor (BDNF), Boston Naming Test (BNT) (Goodglass & Kaplan, 1983), Caregiver Burden Questionnaire (Zarit, Reever, & Bach-Peterson, 1980), Clinical Dementia Rating Scale (CDR) (Morris, 1993), Communication Aphasia Test (CAT): Disability Questionnaire (Swinburn, Porter, & Howard, 2012), Communication Confidence Rating Scale for Aphasia (CCRSA) (Cherney et al., 2011), Catechol-o-methyl transferase (COMT) gene, Dementia Quality of Life Scale (DEMQOL) © Institute of Psychiatry King’s College of London (Smith et al., 2005), Diffusion tensor imaging (DTI), Digit spans forward and backwards (subtest from the Wechsler Adult Intelligence Scale–Fourth Edition (WAIS–IV) (Wechsler, 2008), Disease and life events’ prompts (Nicholas & Brookshire, 1995), Frontotemporal Lobar Degeneration Module (FTLD-MOD) sentence repetition task – extended (Ruch et al., 2022), Global local (Navon, 1977), Hopkins Assessment of Action Naming (HANA) (Breining et al., 2022), Instrumental Activities of Daily Living (IADL) (Lawton & Brody, 1969), Johns Hopkins University (JHU) Dysgraphia Battery (Goodman & Caramazza, 1985), Language Experience and Proficiency Questionnaire (LEAP-Q) (Marian, Blumenfeld, & Kaushanskaya, 2007), Magnetic resonance imaging (MRI), Magnetization-prepared rapid acquisition with gradient echo (MPRAGE), Montreal Cognitive Assessment (MoCA) (Nasreddine et al., 2005), Neurofilament Light chain (NfL), Patient Health Questionnaire (PHQ-9) © Pfizer, 1999 (Kroenke, Spitzer, & Williams, 2001), Philadelphia Naming Test-short form A (PNT) (Walker & Schwartz, 2012), Pittsburgh Sleep Quality Index (PSQI) (Buysse et al., 1989), Progressive Aphasia Severity Scale Questionnaire (PASS) (Sapolsky, Domoto-Reilly, & Dickerson, 2014), Pseudo-Continuous Arterial Spin Labeling (pCASL), Ravens Colored Progressive Matrices (RCPM) (Raven & Raven, 2003), Resilience Evaluation Scale (RES) (van der Meer et al., 2018), Resting-state functional magnetic resonance imaging (RS-fMRI), Rey Auditory Verbal Learning Test (RAVLT) (Rey, 1941), Visual Simon task (Lu & Proctor, 1995), Spatial spans forward and backward (Corsi, 1972), Subject, object, active and passive (SOAP) sentence comprehension (Love & Oster, 2002), Trail Making Test (TMT) (Stuss et al., 2001), Verb and Sentence Test (VAST) (Bastiaanse et al., 2003), Survey of Autobiographical Memory (SAM) © Baycrest, 2012 (Palombo et al., 2013), Wong-Baker FACES^®^ Pain Rating Scale (FACES Scale) (Wong-Baker FACES Foundation, 2022)  * questionnaire filled by both the individual with PPA and their caregiver, independently  ** questionnaire filled only by the caregiver | | | | | | | | |
